# Enhanced glucose metabolism in Tet-deficient mouse embryonic stem cells

**DOI:** 10.3389/freae.2024.1245823

**Published:** 2024-05-06

**Authors:** Yuhan Yang, Maryn Cavalier, Ashley Suris, Kevin Chen, Claire An, Jingyuan Fan, Logan Rivera, Shaohai Fang, Lei Guo, Yubin Zhou, Yun Huang

**Affiliations:** 1 Center for Epigenetics and Disease Prevention, Institute of Biosciences and Technology, Texas A&M University, Houston, TX, United States,; 2 Center for Translational Cancer Research, Institute of Biosciences and Technology, Texas A&M University, Houston, TX, United States,; 3 Department of Translational Medical Sciences, Texas A&M University, Houston, TX, United States

**Keywords:** epigenetics, glucose metabolism, TET, mouse embryonic stem (ES) cells, bioenergetics

## Abstract

Interactions between epigenetics and metabolites play critical roles in regulating the pluripotency and differentiation of embryonic stem cells. Proper glucose metabolism and DNA methylation are essential for orchestrating accurate lineage specification and the normal functions of embryonic stem cells. However, the impact of Ten-eleven Translocation (TET)-mediated DNA methylation modifications on the metabolism of mouse embryonic stem cells (mESCs) remains less well defined. In this study, we investigated the consequences of Tet triple knockout (Tet-TKO) in mESCs and observed notable alterations in glucose metabolism. These changes were marked by enhanced glucose uptake and glycolysis, likely owing to the upregulation of genes critical for glucose metabolism. Furthermore, Tet-TKO mESCs exhibited defects in glucose-dependent differentiation, suggesting that cells with epigenetic defects might display metabolic vulnerability when exposed to external nutritional cues. Collectively, our findings establish the pivotal role of the TET family of dioxygenases in maintaining proper glucose metabolism and safeguarding stem cell lineage specification, thus enhancing our understanding of the intricate interplay between epigenetic modifications and cellular metabolism in stem cells.

## Introduction

Epigenetic and metabolic regulation are fundamental processes that play crucial roles in maintaining proper cellular function. The precise control of epigenetic modifications and metabolite production in stem cells is important for preserving pluripotency and guiding subsequent differentiation (Moussaieff et al., 2015; Shyh-Chang and Ng, 2017; Harvey et al., 2019). For example, elevated glycolysis rates have been shown to contribute to the rapid growth of embryonic stem cells (Tsogtbaatar et al., 2020). Moreover, the reprogramming of somatic cells into induced pluripotent stem cells (iPSCs) is often accompanied by a shift from mitochondrial oxidative phosphorylation (OXPHOS) to glycolysis, highlighting the impact of glycolysis on stem cell identity and lineage commitment (Folmes et al., 2011). Previous studies have shown that the direct and indirect metabolic products of glycolysis, such as acetyl-CoA and acetate, can modulate histone acetylation levels in embryonic stem cells, thus linking metabolite production to epigenetic modifications (Moussaieff et al., 2015). Additionally, high glucose intake has been shown to inhibit AMPK activity, ultimately resulting in reduced phosphorylation and destabilization of Ten-eleven Translocation 2 (TET2) (Wu et al., 2018). TET2 is a key epigenetic enzyme that catalyzes the oxidation of 5-methylcytosine, facilitating DNA demethylation and contributing to transcriptional regulation (Tahiliani et al., 2009). Genetic depletion of TET enzymes (TET1, TET2, and TET3) and loss-of-function mutations therein have been shown to disrupt differentiation and lineage specification of both human and mouse embryonic stem cells. These defects are thought to arise from compromised DNA demethylation and transcriptional regulation (Dawlaty et al., 2014; Li et al., 2022). Despite the association between metabolic and epigenetic regulation, the metabolic characteristics of embryonic stem cells lacking TET enzymes remain largely underexplored, creating a pressing need for further investigations seeking to understand the specific metabolic alterations occurring in these cells and the implications for stem cell function and fate determination.

Metabolites exert profound influence on epigenetic modifications across the mammalian genome. Conversely, epigenetic machinery significantly contributes to the regulation of metabolic networks. For instance, the loss of histone acetylation has been shown to trigger aberrant expression of key genes associated with glucose metabolism (Moussaieff et al., 2015). Using Tet-deficient mouse models, we previously detected aberrant transcriptional activity of genes associated with various metabolic processes during embryonic development (Fang et al., 2019). However, it remains unclear whether these transcriptional alterations produce changes in metabolic outcomes in TET-deficient embryonic stem cells.

In this study, we sought to address this knowledge gap by using Tet-deficient mouse embryonic stem cells (mESCs) as an ideal model system. We observed a pronounced increase in both glucose uptake and glycolysis upon Tet ablation in mESCs. Concurrently, we investigated the impact of Tet deficiency on mESC differentiation by employing an embryoid bodies (EBs) formation assay. Notably, we found that the differentiation defects observed in Tet-deficient cells were dependent on the availability of glucose. Overall, our findings shed new light on the intricate relationship among metabolism, epigenetic regulation, and stem cell function, further highlighting the importance of Tet enzymes in maintaining proper metabolic homeostasis and supporting stem cell function.

## Results

### Tet-deficient mESCs exhibit enhanced glucose metabolism

**FIGURE 1:**
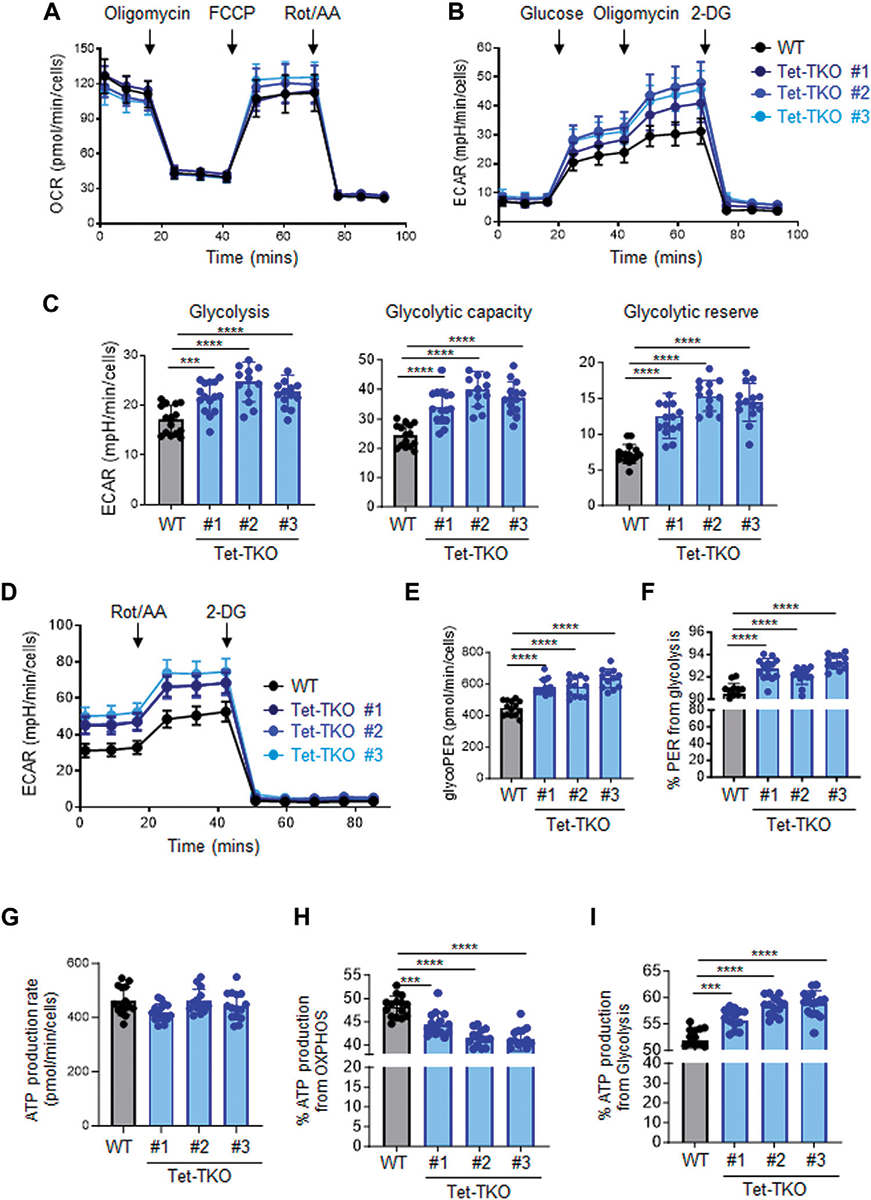
Tet depletion alters glucose metabolism in mESCs. **(A)** Oxygen consumption profiles of WT (black) and Tet-TKO (blue, 3 clones) mESCs before and after sequential addition of oligomycin, FCCP, and rotenone/antimycin. Data are shown as mean ± S.D; *n* = 12/group. **(B)** Rates of extracellular acidification in WT (black) and Tet-TKO (blue, 3 clones) mESCs before and after addition of glucose, oligomycin, 2-Deoxy-d-Glucose (2-DG). Data are shown as mean ± S.D; *n* = 14/group. **(C)** Cellular glycolysis rate, max glycolysis capacity, and reserved glycolytic capacity in WT (black) and Tet-TKO (blue, 3 clones) mESCs, Data are shown as mean ± S.D; n = 14/group. ***p < 0.001, ****p < 0.0001 (Two-tailed Student’s *t*-test). **(D)** Rates of extracellular acidification in WT (black) and Tet-TKO (blue, 3 clones) mESCs before and after addition of rotenone/antimycin and 2-Deoxy-d-Glucose (2-DG). Data are shown as mean ± S.D; *n* = 12/group. **(E,F)** Basal glycolysis analysis **(E)** and percentage of proton efflux rate derived from glycolysis **(F)** in WT (black) and Tet-TKO (blue, 3 clones) mESCs. Data are shown as mean ± S.D; *n* = 12/group. ****p < 0.0001 (Two-tailed Student’s *t*-test). **(G**–**I)** Metabolic flux analysis of total basal ATP production **(G)**, percentage of ATP produced from OXPHOS **(H)** and glycolysis **(I)** in WT (black) and Tet-TKO (blue, 3 clones) mESCs. Data are shown as mean ± S.D; n = 15/group. ***p < 0.001, ****p < 0.0001 (Two-tailed Student’s *t*-test).

To investigate the potential impact of Tet deletion on cellular metabolism, we performed a series of metabolic flux assays using both wildtype (WT) and Tet-triple knockout (Tet-TKO) mESCs. The Tet-TKO mESCs were generated as previously reported utilizing a CRISPR-Cas9-based genome editing approach (Fang et al., 2019). We selected three independent clones of Tet-TKO mESCs and confirmed deletion efficiency through locus-specific Sanger sequencing and western blot analysis (Fang et al., 2019). All three clones showed a complete absence of detectable 5-hydroxymethylcytosine (5 hmC) as a major catalytic product of Tet enzymes. To investigate the impact of Tet deletion on cellular energetics, we assayed oxidative phosphorylation and glycolysis activity in WT and Tet-TKO mESCs by measuring oxygen consumption rates (OCR) and extracellular acidification rates (ECAR), respectively. Mitochondrial stress tests revealed similar oxidative phosphorylation activity between WT and Tet-TKO mESCs (Figure 1A). Tet-TKO mESCs displayed significantly higher ECAR rates than the WT mESCs in a glycolysis stress test, indicating increased glycolysis activity (Figure 1B). Cells were cultured under non-glycolytic conditions to establish a baseline. Subsequent glucose treatment allows calculation of the rate of glycolysis. Oligomycin was added to inhibit oxidative phosphorylation and drive the full capacity of glycolysis, allowing determination of the glycolytic capacity. Finally, cells were exposed to 2-Deoxy-D-glucose (2-DG), a glucose analog that is inefficiently metabolized through glycolysis. The 2-DG treatment evaluates the cellular response to energy demands, enabling the determination of the glycolytic reserve value, or cellular capability to shift energy production towards glycolysis. The Tet-TKO clones exhibited a 1.2 to 2.1-fold increase in the glycolysis rate, glycolytic capacity, and glycolytic reserve ability when cells were successively challenged with glucose, oligomycin, and 2-DG (Figure 1C). Additionally, we quantified the glycolytic rates in WT and Tet-TKO mESCs by exposing cells to rotenone/antimycin A (Rot/AA), followed by 2-DG treatment (Figure 1D). In this assay, Rot/AA is used to inhibit mitochondrial function and measure mitochondria-associated acidification. The use of Rot/AA and 2-DG treatment allows the differentiation between acidification from mitochondrial oxidative phosphorylation and aerobic glycolysis. Our results revealed elevated levels of basal and compensatory glycolysis upon Tet deletion (Figure 1E), suggesting a higher proportion of proton efflux derived from glycolysis in Tet-TKO mESCs compared to WT mESCs (Figure 1F).

ATP, which is derived from both mitochondrial OXPHOS and cytosolic glycolysis, serves as a major energy source to support cell survival and growth. To assess the impact of Tet deletion on ATP production, we calculated ATP production based on the OCR and ECAR data obtained from WT and Tet-TKO mESCs. While the overall basal ATP production rate appeared similar across all groups (Figure 1G), Tet deletion resulted in a higher proportion of ATP being generated from glycolysis compared to OXPHOS (Figures 1H, I). Collectively, these findings indicate that Tet deficiency leads to aberrant glucose metabolism in mESCs.

### Tet-deficient mESCs demonstrate increased glucose uptake

**FIGURE 2:**
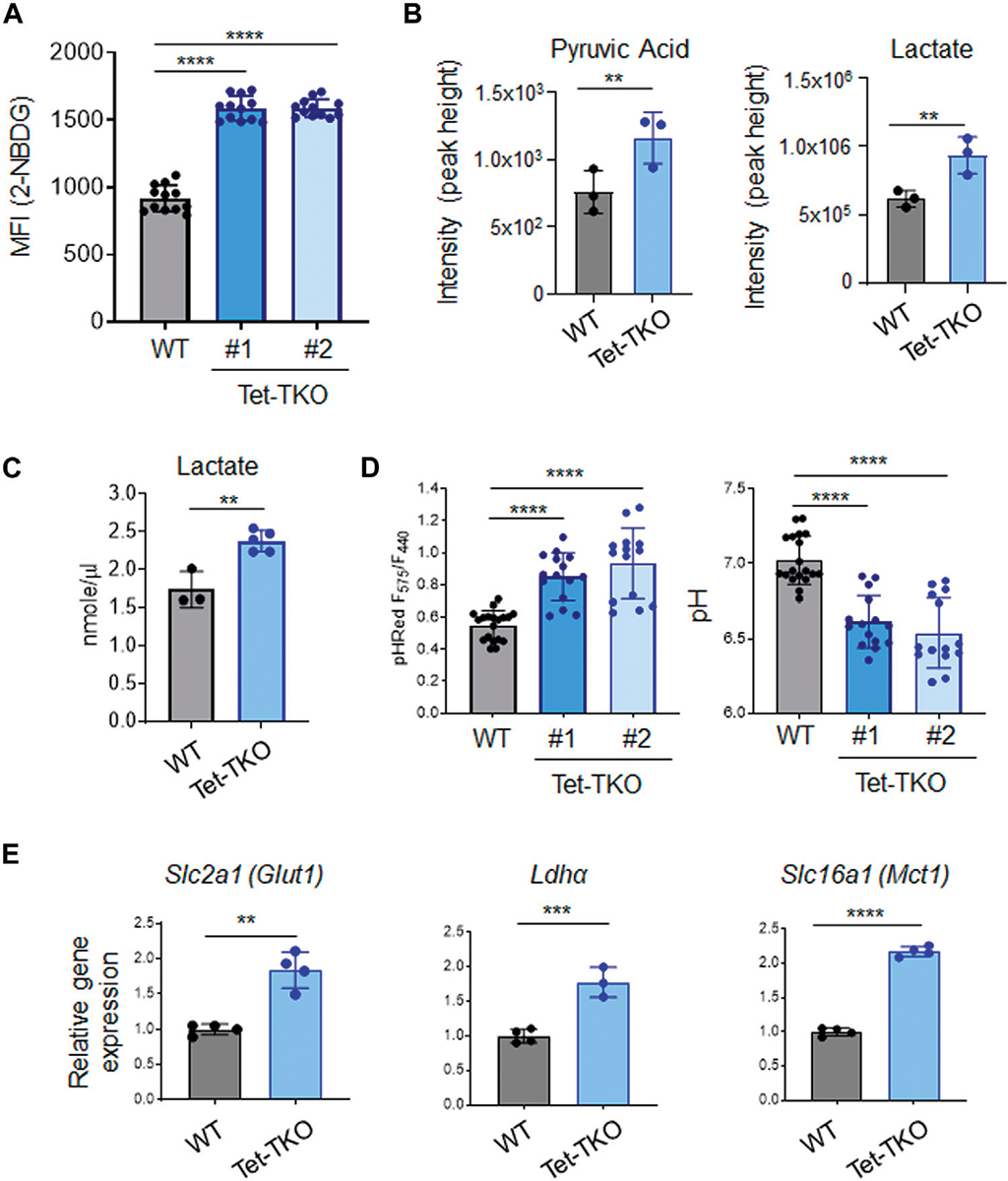
Tet deletion promotes glucose uptake in mESCs. **(A)** Mean fluorescence intensity (MFI) of 2-NBDG in WT (black) and Tet-TKO (blue, 2 clones) mESCs using flow cytometry analysis. Data are shown as mean ± S.D; *n* = 12/group. ****p < 0.0001 (Two-tailed Student’s *t*-test). **(B)** Mass spectrometry analysis of intracellular pyruvic acid and lactate levels in WT (black) and Tet-TKO (blue) mESCs. Data are shown as mean ± S.D; *n* = 3/group. **p < 0.01 (Two-tailed Student’s *t*-test). Data was collected from 3 clones of mESCs. **(C)** Colorimetric lactate assay in WT (black) and Tet-TKO (blue) mESCs. Data are shown as mean ± S.D; *n* = 3–5/group. **p < 0.01 (Two-tailed Student’s *t*-test). Data was collected from 3 clones of mESC. **(D)** Intracellular pH measurement using pHRed by measuring the fluorescence intensity ratio in 575 nm and 440 nm in WT (black) and Tet-TKO (blue, 2 clones) mESCs. The higher ratio suggested lower pH value. Data are shown as mean ± S.D; *n* = 14–19/group, ****p < 0.0001 (Two-tailed Student’s *t*-test). **(E)** The relative gene expression of *Glut1*, *Ldha* and *Mct1* in WT (black) and Tet-TKO (blue) mESCs. Data are shown as mean ± S.D; *n* = 4/group, **p < 0.01, ***p < 0.001, ****p < 0.0001 (Two-tailed Student’s *t*-test). Data was collected from 3 clones of mESC.

High glycolytic flux is a common characteristic observed in various stem cells, including embryonic stem cells. To assess the impact of Tet deletion on glucose uptake, we treated WT and Tet-TKO mESC clones (one and two) with 2-NBDG (2-(N-(7-Nitrobenz-2-oxa-1,3-diazol-4-yl)Amino)-2-Deoxyglucose), a fluorescent glucose analog that allows for direct measurement of glucose uptake using flow cytometry. We observed an approximately 1.7-fold increase in 2-NBDG signals in Tet-TKO mESCs compared to the WT group (Figure 2A), suggesting enhanced glucose uptake following Tet deletion. In parallel, we employed mass spectrometry analysis to measure the intracellular pyruvate and lactate levels in the same cells. Consistent with 2-NBDG uptake results, we found elevated levels of both pyruvate and lactate in Tet-TKO mESCs compared to WT cells (Figure 2B). The increase of lactate uptake is also observed in cells lacking SIRT6, an NAD + -dependent histone deacetylase (Zhong et al., 2010). The increased intracellular lactate level in Tet-TKO cells was further independently validated by lactate colorimetric assay (Figure 2C). Considering that elevated intracellular lactate has been reported to alter the intracellular pH level (Miyazawa et al., 2022), we introduced a ratiometric pH sensor, GW1-pHRed, into WT and Tet-TKO mESCs. Tet-TKO mESC showed a more prominent increase in the fluorescence intensity ratio of the pH sensor, indicating a lower pH in the cytosol (Figure 2D). Overall, these results indicate enhanced glucose uptake, higher levels of pyruvic acid and lactate, and disrupted intracellular pH regulation in Tet-TKO mESCs, supporting the role of Tet genes in modulating stem cell metabolism.

To gain further insights into the mechanism underlying increased glucose uptake in Tet-TKO mESCs, we examined the transcription of key genes involved in glucose uptake using real-time quantitative PCR (qPCR). Our analysis revealed a significant upregulation of *Slc2a1* (Glut1*), Ldha, and Slc16a1* (Mct1) genes in Tet-TKO clones compared to WT cells (Figure 2E). Glucose transporter 1 (*Glut1)* is a transporter protein directly involved in the uptake of glucose into cells (Olson and Pessin, 1996). Lactate dehydrogenase A (*Ldha)* is an enzyme involved in the metabolism of L-lactate and pyruvate (Chung et al., 1985). Monocarboxylate transporter 1 *(Mct1)* encodes a proton-coupled symporter called that facilitates lactate transportation (Garcia et al., 1994). Collectively, these findings provide valuable insights into the molecular changes associated with Tet deletion and the potential mechanisms underlying the observed increase in glucose uptake and metabolism in Tet-deficient mESCs.

### Tet depletion has minimal impact on proliferation and pluripotency in mESCs

**FIGURE 3:**
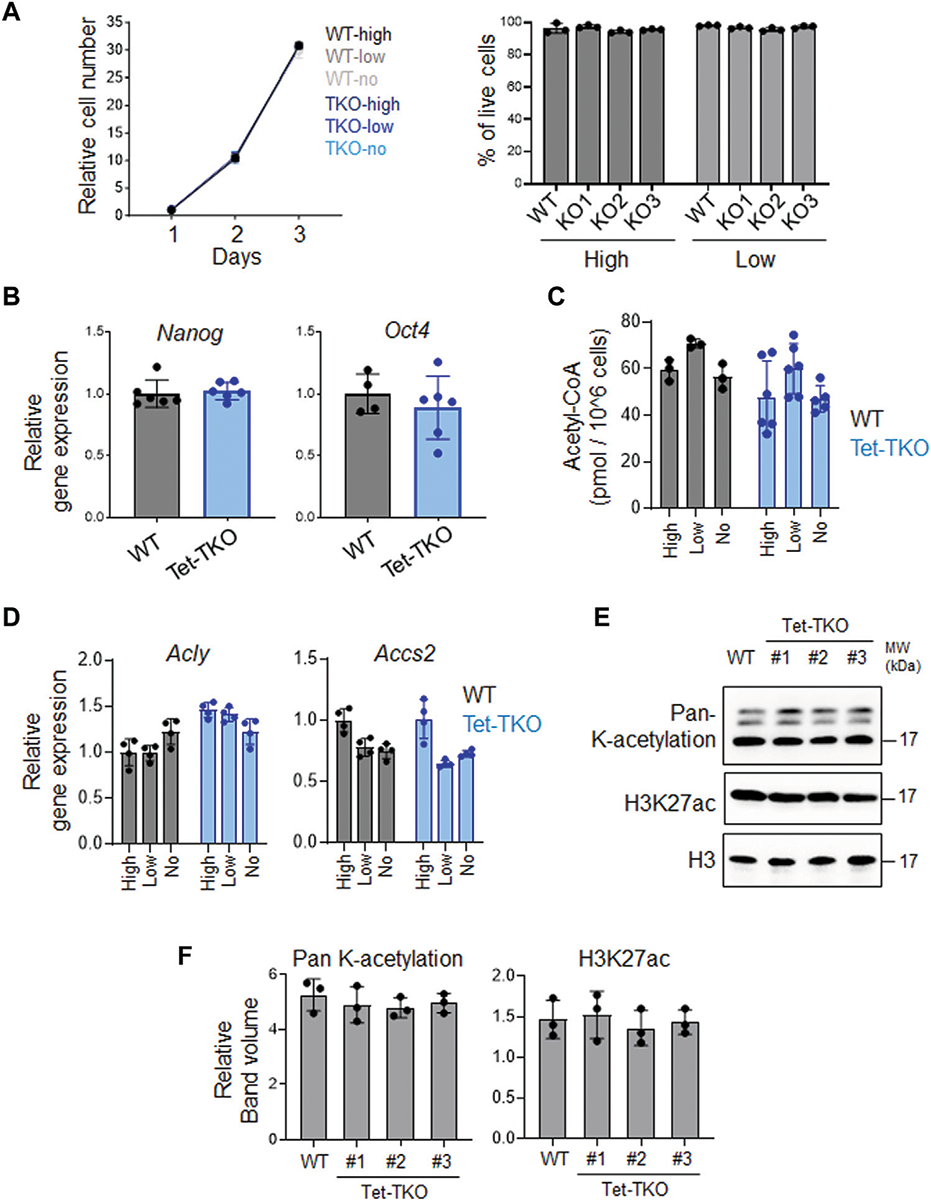
Tet depletion has minimal impact on proliferation and pluripotency in mESCs. **(A)** Growth curve (left) and viability (right) of WT and Tet-TKO mESCs cultured in different amounts of glucose. Data are shown as mean ± S.D; *n* = 3/group. **(B)** The relative gene expression of *Nanog* and *Oct4* in WT (black) and Tet-TKO (blue) mESCs. Data are shown as mean ± S.D; *n* = 3/group. Data was collected from 3 clones of mESCs. **(C)** The intracellular acetyl-CoA level in WT (black) and Tet-TKO (blue) mESCs cultured in different amounts of glucose. Data are shown as mean ± S.D; *n* = 3/group. Data was collected from 3 clones of mESC. **(D)** The relative gene expression of *Acly* and *Accs2* in WT (black) and Tet-TKO (blue) mESCs cultured in different amounts of glucose. Data are shown as mean ± S.D; *n* = 4/group. Data was collected from 3 clones of mESC. **(E,F)** Representative image **(E)** and quantification **(F)** of western-blot analysis of total histone lysine acetylation and H3K27ac level in WT and Tet-TKO (3 clones) mESCs. Total histone H3 was used as loading control.

To investigate whether elevated glucose intake and metabolism in Tet-TKO mESCs alter cellular function, we measured cell proliferation and viability under different glucose concentrations (high: 4.5 g/L, low: 1.0 g/L, no: 0 g/L). No significant difference in cell growth was observed between WT and Tet-TKO mESCs under these conditions (Figure 3A). Additionally, qPCR analysis revealed no significant changes in the expression levels of *Nanog* and *Oct4*, key genes involved in stem cell pluripotency (Figure 3B). These data suggest that increased glucose metabolism resulting from Tet deficiency has minor impacts on the proliferation and pluripotency of mESCs.

Given that increased glucose levels can lead to upregulation of acetyl-CoA and alter histone acetylation, we measured the intracellular acetyl-CoA level and the transcription of key genes involved in acetyl-CoA metabolism (Figures 3C, D). Our data suggests that increased intracellular glucose levels in Tet-TKO mESCs do not exert significant effects on acetyl-CoA levels or the expression of *Acly* and *Accs2*, two key genes involved in acetyl-CoA production (Wellen et al., 2009; Mews et al., 2017). Furthermore, the histone acetylation level remains unaffected in Tet-deficient mESCs (Figures 3E, F).

### Tet deletion increases sensitivity to glucose deficiency in mESCs during their differentiation

**FIGURE 4:**
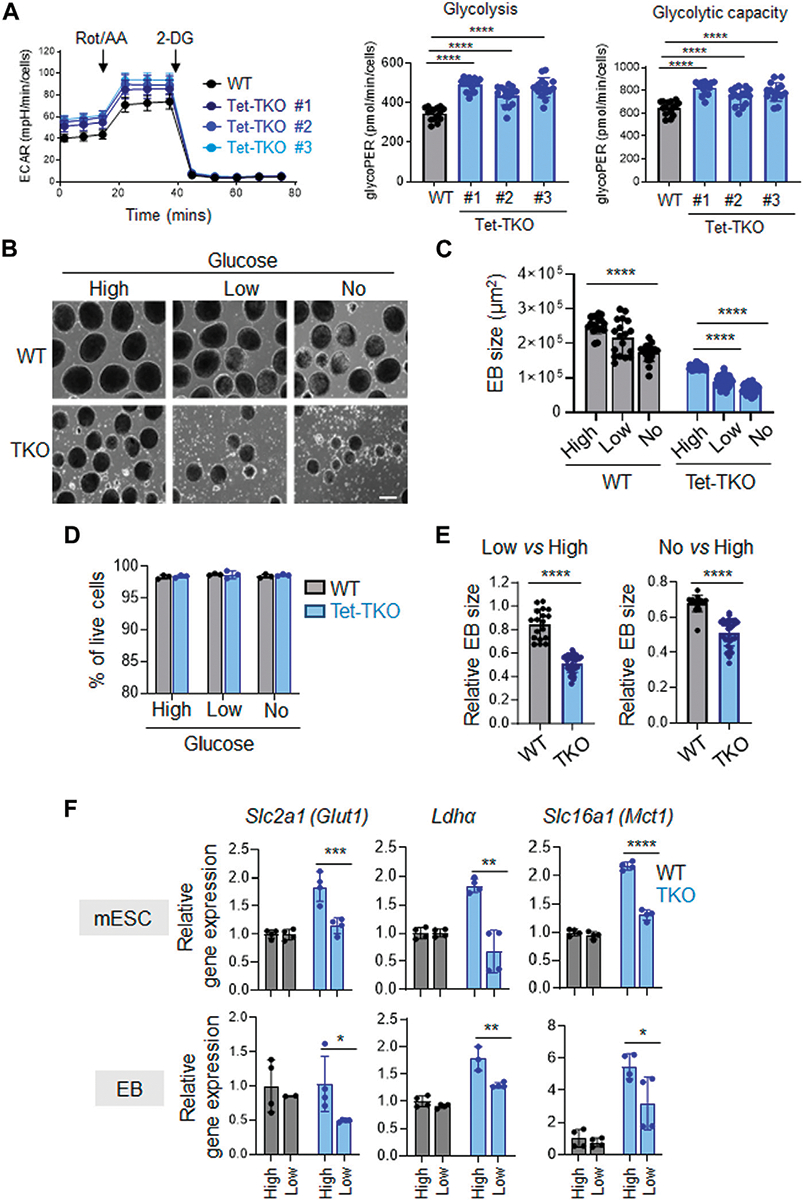
Tet-deficient mESCs are more sensitive to glucose deficiency than WT mESCs **(A)** Rates of extracellular acidification in embryoid bodies (EBs) differentiated from WT (black) and Tet-TKO (blue, 3 clones) mESC before and after addition of rotenone/antimycin and 2-Deoxy-d-Glucose (2-DG). Cellular glycolysis rate and glycolysis capacity changes in EBs differentiated from WT (black) and Tet-TKO (blue, 3 clones) mESCs, Data are shown as mean ± S.D; *n* = 14/group, ****p < 0.0001 (Two-tailed Student’s *t*-test). **(B)** Representative images of EBs differentiated from WT and Tet-TKO mESCs (clone 1) cultured in high, low and no glucose medium. Scale bar: 300 μm. **(C)** The absolute EB size in WT and Tet-TKO groups cultured under high, low, or no glucose conditions. Data are shown as mean ± S.D; *n* = 18–33/group, ****p < 0.0001 (Two-tailed Student’s *t*-test). Data were collected from three independent clones of mESCs. **(D)** The EBs under corresponding conditions were dissociated 5 days after differentiation and then stained with 7-AAD. Flow cytometry analysis was performed to evaluate viability. Data are shown as mean ± S.D; *n* = 3, (Two-tailed Student’s *t*-test). Data were collected from three independent clones of mESCs. **(E)** Quantification of relative changes of EB sizes between low vs. high glucose and no vs. high glucose conditions in WT or Tet-TKO groups. Data are shown as mean ± S.D; *n* = 18–33/group, ****p < 0.0001 (Two-tailed Student’s *t*-test). Data was collected from 3 clones of mESC. **(F)** The relative gene expression of *Glut1*, *Ldha*, and *Mct1* in undifferentiated mESCs (top) and differentiated EBs (bottom) in WT (black) and Tet-TKO (blue) groups cultured in different amounts of glucose. Data are shown as mean ± S.D; *n* = 3–4/group. *p < 0.05, **p < 0.01, ***p < 0.001, ****p < 0.0001 (Two-tailed Student’s *t*-test). Data was collected from three independent clones of mESCs.

Next, we conducted additional investigations to determine the impact of different glucose concentrations on the differentiation capability of Tet-TKO mESCs. To assess this, we performed embryoid body (EB) formation assays using WT and Tet-TKO mESCs at varying glucose levels: high (4.5 g/L), low (1.0 g/L), and no glucose (0 g/L). Similar to the undifferentiated cells, the differentiated Tet-TKO embryoid bodies exhibited significantly higher ECAR than that of WT embryoid bodies in a glycolytic rate test (Figure 4A). Consistent with previous research (Koh et al., 2011; Fang et al., 2022), Tet-TKO mESCs showed a significant reduction in the ability to form embryoid bodies under all conditions in a dose-dependent manner, impacting both the size and number of EBs without affecting cell viability (Figures 4B–D). Interestingly, WT mESCs showed only minor changes in response to reduced glucose conditions (Figures 4B, C). In fact, the relative EB size of Tet-TKO mESCs cultured under high glucose conditions was significantly lower than the EB size of WT-TKO mESCs under low and no glucose conditions (Figure 4E). To gain further insights, we measured the expression of genes crucial for glucose metabolism in differentiated WT and Tet-TKO cells under different glucose concentrations. Similar to undifferentiated mESCs, Tet-TKO embryoid bodies continued to exhibit higher expression of genes involved in glucose metabolism, such as *Glut1, Ldha*, and *Mct1*, under high-glucose culture conditions (Figure 4F). Interestingly, a significant reduction in the expression of these genes was detected in Tet-TKO, but not WT, embryoid bodies under reduced glucose culture conditions compared to the high glucose culture condition (Figure 4F). In summary, these findings indicate that Tet-deficient mESCs rely more heavily on glucose levels compared to WT cells during differentiation.

## Discussion

In our study, we delineated the metabolic characteristics of Tet-TKO mESCs, noting an augmentation in glucose metabolism—specifically in glucose uptake and glycolysis—in Tet-deficient cells. This condition led to elevated lactate production and decreased intracellular pH levels. Previous transcriptomic analyses using Tet-deficient mESCs and tissue-specific Tet-deficient mouse models have identified altered expression of genes related to metabolism (Dawlaty et al., 2014; Fang et al., 2019). However, the direct impact of these alterations on the metabolic functionality in Tet-deficient stem cells remained ambiguous. Our results provide compelling evidence to demonstrate alterations in glucose metabolism illustrated by enhanced glycolysis and glucose uptake in Tet-deficient mESCs. It is worth mentioning that Tet-deficient cells may not be solely confined to glucose metabolic defects. The metabolic irregularities might vary based on the cell line and the biological system employed, as different systems rely on diverse metabolic pathways. Considering the crucial role of glucose in regulating pluripotency and proliferation in mESCs, our study has uncovered a prominent glucose metabolic signature in Tet-TKO mESCs.

Although Tet depletion has minimal effects on glucose metabolism in undifferentiated WT mESCs, we observed prominent changes in Tet-TKO mESCs. Moreover, the differentiation effects observed in Tet-TKO mESCs were highly reliant on the glucose level in the culture medium, in contrast to WT mESCs, which exhibited consistent differentiation capabilities across various glucose concentrations. This indicates that, unlike WT mESCs, Tet-TKO mESCs rely heavily on extracellular glucose levels during the differentiation process. In light of the critical roles of both epigenetic and metabolic regulators in supporting normal development, our findings suggest that epigenetic defects during early development, as exemplified by Tet loss-of-function, may lead to metabolic vulnerability in response to variations in nutritional availability. Our findings indicate that Tet-TKO mESCs exhibit increased expression of genes involved in glucose metabolism, which might account for the observed enhancement in glycolysis. Although the Tet enzymes are known as DNA methylation dioxygenases and are usually associated with transcriptional activation, there have also been reports of Tet proteins exerting a catalytic-independent role to suppress gene expression by partnering with HDAC2 (Zhang et al., 2015; Fuster et al., 2017). Further studies are needed to pinpoint the underlying molecular mechanism and delineate the catalytic-dependent or independent functions of TET proteins in regulating glucose metabolism. Overall, our findings underscore the importance of functional interplays between epigenetic modifications and metabolic processes in maintaining optimal cellular function and developmental outcomes.

## Materials and methods

### mESC culture under various glucose levels

Mitomycin C treated mouse embryonic fibroblasts (MEF) were used as feeder cells for mESCs culture in this study. Normal culture medium is the Knockout Dulbecco’s Modified Eagle’s Medium (Gibco) supplemented with 15% fetal bovine serum (Omega), 1.0% penicillin-streptomycin (Gibco), 0.1 mM non-essential amino acids (Gibco), 0.1 mM 2-mercaptoetanol (Sigma), and 103 U/mL of leukemia inhibitory factor (LIF; Millipore). Various glucose treatment mediums consisted of DMEM without glucose supplemented with 15% fetal bovine serum (Omega), 1% penicillin-streptomycin (Gibco), 0.1 mM non-essential amino acids (Gibco), 0.1 mM 2-mercaptoethanol (Sigma), 103 U/mL of leukemia inhibitory factor (LIF; Millipore), and D-(+)-glucose solution 45% at varying concentrations (high: 4.5 g/L; low: 1.0 g/L; and no: 0 g/L; Corning).

### Embryoid body (EB) formation

EB formation is performed using the hanging drop culture method. mESCs are suspended into single cells and then resuspended in EB formation media (mESC culturing medium without LIF). The cells are then suspended in a dish pre-coated with 0.2% gelatin and incubated at 37°C with 5% CO2 for 30 min to remove MEF. mESCs are resuspended at 500 cells per 20 μL (20 μL/drop) containing varying glucose concentrations and inoculated onto the lid of the tissue culturing dish. The lid is then inverted and placed on top of the dish containing 15 mL of PBS and cultured for 5 days. Afterward, the EBs are collected for further analysis. For the cell viability assay, the EBs are washed once with PBS and then trypsinized into single cells. The 7-AAD Viability Staining Solution (Biolegend) followed by flow cytometry analysis is performed to check the cell viability.

### Seahorse analysis

Mouse ESCs were seeded in XF96 cell culture microplates (Agilent) coated with 1.5% fibronectin at a density of approximately 20,000 cells per well in normal culture medium, and then incubated overnight. The following day, the Seahorse Glycolytic Rate Assay, Seahorse Glycolysis Stress Test, Seahorse Mito Stress Test, or ATP Real-Time Rate Assay were performed on the XF96 Analyzer (Agilent) according to the manufacturer’s instructions. Seahorse results were normalized to cell counts. The normalized Seahorse data were analyzed using Wave, the software for the XF Analyzer from Agilent.

### RNA extraction and real-time quantitative PCR (qPCR)

Mouse ESCs were harvested, and DNA-free RNA was isolated using the NucleoSpin RNA Plus kit (MACHEREY-NAGEL), as per the manufacturer’s manual. Purified total RNA (500 ng) was reverse transcribed into cDNA using the PrimeScript RT Master Mix (TaKaRa). Real-time quantitative PCR was performed on a QuantStudio3 (Thermo Fisher Scientific) instrument using the 2X Universal SYBR Green Fast qPCR Mix (ABclonal). A three-step cycling program was employed, which included an initial denaturation at 95°C for 5 min, followed by 30 cycles of denaturation at 95°C for 10 s, annealing at 60°C for 20 s, and extension at 72°C for 30 s. The mRNA expression of the target gene was normalized to 18S. All the primers were synthesized by Integrated DNA Technologies.

### Protein extraction and Western-blot analysis

mESCs were rinsed with PBS twice and lysed in a radio-immunoprecipitation assay (RIPA) buffer (150 mM NaCl, 1% TritonX-100, 0.1% SDS, 0.5% Sodium deoxycholate, 50 mM Tris-HCl pH8.0), supplemented with Xpert Protease Inhibitor Cocktail Solution (100X) (GenDEPOT) on ice for 15 min. Cell debris was removed by centrifugation at 12,000 × g at 4°C for 15 min. For histone extraction, mESCs were rinsed with PBS twice and lysed in Triton extraction buffer (TEB: PBS containing 0.5% Triton X-100 (v/v), 2 mM phenylmethyl-sulfonylfluoride (PMSF), 0.02% (w/v) NaN3) on ice for 15 min. The lysate was then centrifuged at 12,000 × g at 4°C for 15 min, and the supernatant was removed and discarded. The pellets were then washed with TEB twice and resuspended in 0.2 N HCl overnight at 4°C. The lysates were then centrifuged at 12,000 × g at 4°C for 15 min, and the supernatant was collected for western blot analysis. The cell lysate was loaded onto 10% or 14% SDS-PAGE gels after mixing with SDS loading buffer (100 mM Tris-HCl, 4% SDS, 0.2% bromophenol blue, 20% glycerol, 200 mM DTT, pH 6.8) and denaturing at 100°C for 10 min. Proteins were transferred onto Nitrocellulose membranes (Millipore) and blocked in 5% Bovine Serum Albumin (GenDEPOT) for 1 h at room temperature. Membranes were then incubated with the primary antibodies listed below at 4°C overnight, followed by incubation with a secondary antibody at room temperature for 1 h. West-Q Pico Dura ECL Solution (GenDEPOT) was used for development, and the antigen–antibody complexes were detected using the ChemiDoc Imaging system (Bio-Rad). Antibodies used were: Anti-rabbit IgG, HRP-linked Antibody (Cell Signaling #7074), anti-rabbit IgG, HRP-linked Antibody (Cell Signaling #7076), anti-Histone H3 (Abcam, ab1791), anti-Histone H3 (acetyl K27) (Abcam, ab4729), anti-β-Actin (ABclonal, AC038), anti-GAPDH (ABclonal, AC027), and anti-acetyl lysine (Sigma-Aldrich, AB3879).

### Assays to measure lactate and acetyl-CoA

mESCs were seeded in a 12-well cell culture plate with a final volume of 1.5 mL of normal culture medium per well, and then incubated under standard cell culture conditions overnight. Subsequently, the cells were rinsed with PBS and incubated with normal culture medium for 2 h. Cell pellets were then collected and tested for lactate levels following the manufacturer’s instructions for the Lactate Assay Kit (MAK064, Sigma-Aldrich) or for acetyl-CoA levels following the instructions for the Acetyl-Coenzyme A Assay Kit (MAK039, Sigma-Aldrich).

### Intracellular pH measurement

The GW1-pHRed (Tantama et al., 2011) plasmid was purchased from Addgene (#31473). mESCs were transfected with pHRed using the iMfectin DNA transfection reagent (Gendepot). The pHRed signal was captured using the W1-Yokogawa/Nikon Live cell Imaging Spinning Disk confocal microscope. pHRed was excited using dual peaks at 560 and 430 nm, and its emission peak was at 610 nm. ImageJ was utilized for the measurement and quantification of histological data.

### Glucose uptake assay

A FACS-based glucose uptake assay was conducted according to a previously published method (Dong and Alahari, 2018). Briefly, mESCs were seeded in a 24-well cell culture plate with a final volume of 1.0 mL of normal culture medium per well, and then incubated under standard cell culture conditions overnight. Thereafter, the cells were rinsed with PBS and incubated with 1.0 mL of culture media with varying glucose concentrations for 24 h. Next, the cells were rinsed with PBS and incubated with 1.0 mL of culture media containing various glucose concentrations, supplemented with 100 μM 2-NBDG for 2 h. The cell pellets were then collected and kept in ice-cold FACS buffer (PBS with 2% FBS) for analysis on a BD Biosciences LSRII analytical flow cytometer. The mean fluorescence intensities were analyzed using the FlowJo software.

### Cell viability assay

A FACS-based cell viability assay was conducted according to the manufacturer’s instructions. Briefly, LIVE/DEAD Fixable Violet stain (Invitrogen) was diluted by adding 50 μL of DMSO to the vial. Mouse ESCs were seeded in a 24-well cell culture plate with a final volume of 1.0 mL of normal culture medium per well, and then incubated under standard cell culture conditions overnight. Thereafter, the cells were rinsed with PBS and incubated with 1.0 mL of culture media with varying glucose concentrations for 24 h. The cell pellets were then resuspended in 1 mL of FACS buffer with 1 μL of diluted live/dead dye, followed by a 30-min incubation at room temperature. The cells were washed twice with FACS buffer before analysis on a BD Biosciences LSRII analytical flow cytometer. The gating and analysis of the percentages of viable or dead cells were performed using the FlowJo software.

## Data Availability

The original contributions presented in the study are included in the article/Supplementary material, further inquiries can be directed to the corresponding authors.
